# Epigenetic DNA Methylation of *EBI3* Modulates Human Interleukin-35 Formation via NFkB Signaling: A Promising Therapeutic Option in Ulcerative Colitis

**DOI:** 10.3390/ijms22105329

**Published:** 2021-05-19

**Authors:** Alexandra Wetzel, Bettina Scholtka, Fabian Schumacher, Harshadrai Rawel, Birte Geisendörfer, Burkhard Kleuser

**Affiliations:** 1 Institute of Nutritional Science, University of Potsdam, 14558 Nuthetal, Germany; alwetzel@uni-potsdam.de (A.W.); scholtka@uni-potsdam.de (B.S.); rawel@uni-potsdam.de (H.R.); geisendoerfer@uni-potsdam.de (B.G.); 2 Institute of Pharmacy, Freie Universität Berlin, 14195 Berlin, Germany; fabian.schumacher@fu-berlin.de

**Keywords:** decitabine, DNMT inhibitor, EBI3, inhibitory cytokines, interleukin-35, TNFα, Ulcerative colitis

## Abstract

Ulcerative colitis (UC), a severe chronic disease with unclear etiology that is associated with increased risk for colorectal cancer, is accompanied by dysregulation of cytokines. *Epstein–Barr virus-induced gene 3 (EBI3)* encodes a subunit in the unique heterodimeric IL-12 cytokine family of either pro- or anti-inflammatory function. After having recently demonstrated that upregulation of *EBI3* by histone acetylation alleviates disease symptoms in a dextran sulfate sodium (DSS)-treated mouse model of chronic colitis, we now aimed to examine a possible further epigenetic regulation of *EBI3* by DNA methylation under inflammatory conditions. Treatment with the DNA methyltransferase inhibitor (DNMTi) decitabine (DAC) and TNFα led to synergistic upregulation of *EBI3* in human colon epithelial cells (HCEC). Use of different signaling pathway inhibitors indicated NFκB signaling was necessary and proportional to the synergistic *EBI3* induction. MALDI-TOF/MS and HPLC-ESI-MS/MS analysis of DAC/TNFα-treated HCEC identified IL-12p35 as the most probable binding partner to form a functional protein. EBI3/IL-12p35 heterodimers (IL-35) induce their own gene upregulation, something that was indeed observed in HCEC cultured with media from previously DAC/TNFα-treated HCEC. These results suggest that under inflammatory and demethylating conditions the upregulation of *EBI3* results in the formation of anti-inflammatory IL-35, which might be considered as a therapeutic target in colitis.

## 1. Introduction

Ulcerative colitis (UC) is a severe multifactorial disease with a major impact on quality of life and is associated with possibly acute life-threatening complications such as massive blood loss or intestinal perforation due to toxic megacolon [1]. Because of the chronic inflammatory process, patients with UC have an increased risk of developing colorectal cancer [2,3,4]. The precise etiology is still not clear. Besides genetic susceptibility, a role of the gut microbiome, influenced by diet, environmental factors, drugs, etc., on the pathogenesis of IBD is discussed, since dysbiosis leads to widespread alteration of the intestinal environment and destruction of the intestinal barrier [5,6,7,8]. UC is associated with a dysregulation of pro- and anti-inflammatory cytokines. These immunomodulating signaling molecules are involved in autocrine, paracrine, and endocrine processes and play a part in inflammation. The interleukin (IL)-12 cytokine family comprises heterodimeric proteins composed of interchangeable α- and β-subunits: IL-12 (IL-12p35/IL-12p40), IL-23 (IL-23p19/IL-12p40), IL-27 (IL-27p28/Epstein–Barr virus-induced gene 3 (EBI3)), IL-35 (IL-12p35/EBI3), and IL-39 (IL-23p19/EBI3). This interchangeability ensures high flexibility in responding to inflammatory stimuli, quickly switching pro- or anti-inflammatory effects. The involvement of IL-12 cytokines has been described in multiple chronic inflammatory diseases, including UC [9,10,11], uveitis [12,13], multiple sclerosis [13], and rheumatoid arthritis [14]. IL-12, IL-23, and IL-39 are pro-inflammatory, whereas IL-35 is anti-inflammatory. IL-27 has both pro- and anti-inflammatory effects. By means of knockout mouse models, a deficiency in the IL-35 subunit Ebi3 was shown to play a role in experimental colitis manifestation [15,16]. It is known that IL-35 is upregulated in acute inflammation and acts to suppress the proliferation of activated CD4^+^ CD25^−^ T cells as well as the IL-4 production from these. Notably, IL-35 levels are decreased in chronic inflammatory diseases such as allergic asthma [17] and psoriasis [18].

Since UC is a disease with a relapsing–remitting clinical course, epigenetic regulation mechanisms of *EBI3* in colitis appeared possible. Indeed, our group recently demonstrated that inflammation-induced EBI3 expression in human colon epithelial cells (HCEC) could be further enhanced by histone deacetylase inhibitors (HDACi) [19]. Moreover, HDACi treatment of a murine model of dextran sulfate sodium (DSS)-induced chronic colitis improved colitis symptoms: diarrhea, weight loss, spleen weight, CD3^+^ T-lymphocyte invasion, and survival in wild-type mice. Importantly, these were exacerbated in Ebi3^−/−^ mice, suggesting that Ebi3 largely functions as part of the anti-inflammatory IL-35 in intestinal epithelia. Indeed, there was evidence that specific downstream targets of IL-35, such as *Interferon gamma* and *IL-10*, were also induced by HDACi treatment. By contrast, specific downstream targets of IL-39 activity, such as *IL-17a*, *B-cell lymphoma-2*, and *B-cell lymphoma-extra large*, were not upregulated.

Since histone modifications are often associated with further epigenetic processes [20], we investigated whether DNA methylation performs an additional role in the regulation of *EBI3* under inflammatory conditions such as seen in UC. In this context, we especially focused on the intracellular signaling pathways mediating the *EBI3* induction and on potential binding partners. Two previous studies with nonhuman material could lead to the assumption that DNA methylation might be involved in *EBI3* regulation. Li et al. assessed the ratio of methyl group donor S-adenosyl-methionine versus the acceptor S-adenosylhomocysteine as a measure of cellular methylation status in mice. Thus, tissue mRNA expression levels of murine *IL-12p35* and *Ebi3* tended to increase with decreasing tissue S-adenosyl-methionine/S-adenosylhomocysteine ratios, suggesting hypomethylation might induce IL-35 expression [21]. Wang et al. performed genome-wide DNA methylation analysis on polyinosinic–polycytidylic acid-treated pig peripheral blood mononuclear cells via methylated DNA immunoprecipitation sequencing and noted *Ebi3* expression was impacted by methylation status [22].

Here, we show inhibition of DNA methylation increased EBI3 expression directly in intestinal epithelial cells and give evidence supporting the subsequent formation of IL-35. Thus, DNA methylation appears to act alongside histone acetylation in the epigenetic regulation of *EBI3* and directed formation of the anti-inflammatory cytokine IL-35.

## 2. Results

### 2.1. Synergistic Action of TNFα and Inhibition of DNA Methylation Greatly Increases Inflammation-Induced EBI3 Expression

**Figure 1:**
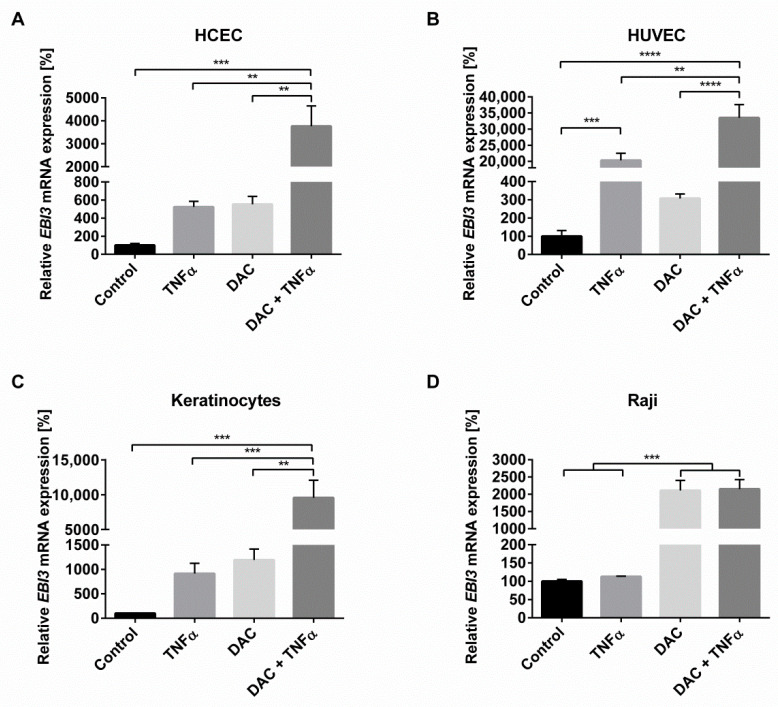
Inhibition of DNA methylation increases *Epstein–Barr virus-induced gene 3 (EBI3)* mRNA expression in human cells from various organs. Human colon epithelial cells (HCEC) (**A**), human umbilical vein endothelial cells (HUVEC) (**B**), primary keratinocytes (**C**), and Raji cells (**D**) were treated with decitabine (DAC) (10 µM) for 48 h (**A**,**B**,**D**) or 24 h (**C**) with or without the addition of TNFα (20 ng/mL) for the final 24 h. The relative quantification of *EBI3* mRNA levels was measured via quantitative reverse transcription polymerase chain reactions (RT-qPCR). *Hydroxymethylbilane synthase* (*HMBS*) served as the reference gene. Data are presented as mean ± standard error of the mean (SEM) from three independent experiments. Statistical analysis was performed using one-way ANOVA and Tukey’s post hoc test (** *p* < 0.01; *** *p* < 0.001; **** *p* < 0.0001).

Based on recent work in which the IL-35 cytokine subunit *EBI3* was shown to be epigenetically regulated by histone acetylation [19], we set out to investigate whether a supplementary epigenetic regulation mechanism via DNA methylation also occurs. The reversal of epigenetic gene silencing is possible by removing promoter hypermethylation. Therefore, the impact of the DNA methyltransferase inhibitor (DNMTi) decitabine (DAC) on inflammation-induced induction of *EBI3* was examined in HCEC, a cell line generated from normal human colon epithelium. Stimulation with either DAC or the inflammatory stimulant TNFα induced a significant 5–6-fold increase in *EBI3* mRNA expression as compared with the vehicle control (Figure 1A). Notably, the combination of both treatments resulted in a significant enhancement from 24 h onwards, peaking with a 40-fold increase at 48 h (Supplementary Figure S1), suggestive of a synergistic mechanism.

To evaluate if the *EBI3*-inducing effect via inhibition of DNA methylation, especially under inflammatory conditions, is a general mechanism, other cells from different origins were likewise examined. Hereby, the synergistic effect was also observed in the endothelial cells human umbilical vein endothelial cells (HUVEC) (Figure 1B) and in primary epidermal human keratinocytes (Figure 1C), indicating a general mechanism of *EBI3* induction after combined TNFα and DAC stimulation. The B-lymphocytic Raji cell lines, derived from Burkitt’s lymphoma, that did not possess a membrane bound TNFα receptor, were unaffected by TNFα treatment (Figure 1D). Together, these results indicate that *EBI3* is regulated via epigenetic DNA methylation in the presence of TNFα signaling. To further evaluate the *EBI3*-inducing effect evoked by DAC and TNFα in HCEC, DAC was replaced with 5-azacytidine (AZA), another substance with a demethylating function [23]. Stimulation of HCEC with AZA or with AZA and TNFα also resulted in increased *EBI3* levels relative to the vehicle control, but to a lesser extent than DAC (Supplementary Figure S2). In total, AZA exerted an over-additive effect on inflammation-induced *EBI3* expression in HCEC.

### 2.2. Influence of Inflammatory Stimulation on DNA Methylation Processes in HCEC

In order to characterize the impact of DNA methylation status on HCEC response to inflammatory stimuli, the time-dependent mRNA expressions of DNMT enzymes in HCEC were assessed via quantitative reverse transcription polymerase chain reaction (RT-qPCR). Following stimulation with TNFα, *DNMT1* mRNA levels decreased slowly to about 70% of the vehicle control after 72 h, while *DNMT3a* and *DNMT3b* levels demonstrated a fast decrease within 8 and 2 hours, respectively, after TNFα treatment (Supplementary Figure S3A). Additionally, the TNFα-induced changes in the expression of the demethylating Ten-eleven translocation (TET) enzymes were investigated. *TET1* mRNA decreased following short- and long-term stimulation with TNFα, while *TET2* was unaffected (Supplementary Figure S3B). Interestingly, although *TET3* levels had increased significantly after 4 h of TNFα stimulation (41% compared to the vehicle control), they then dropped below control levels at 24 h and 48 h of TNFα stimulation. Basal expressions of *DNMT1* and *TET3* were significantly higher than that of their other respective isoforms (Supplementary Figure S3C,D).

**Figure 2:**
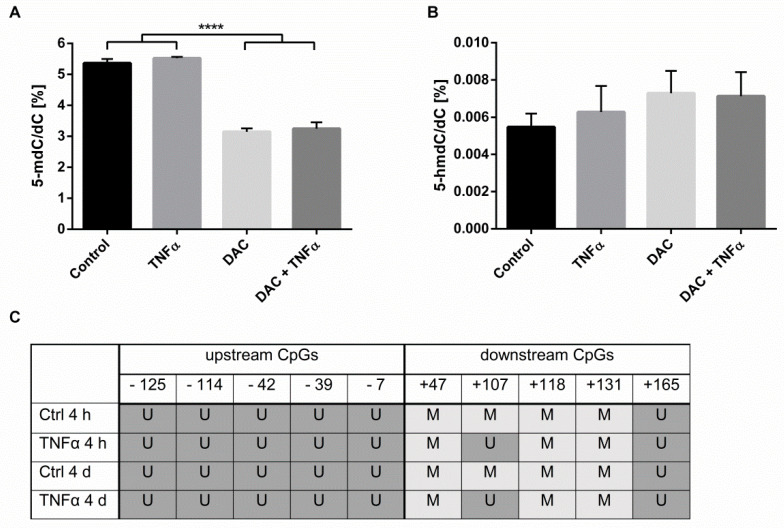
EBI3 gene-specific methylation, but not global methylation, in HCEC is inflammation-dependent. (**A**,**B**) HCEC cells were treated with DAC (10 µM) for 48 h with or without the addition of TNFα (20 ng/mL) for the final 24 h. The genomic levels of 5-mdC/dC (**A**) and 5-hmdC/dC (**B**) were then analyzed via LC-MS/MS. Data are shown as mean ± SEM from three independent experiments. Statistical analysis was performed using one-way ANOVA and Tukey’s post hoc test (**** *p* < 0.0001). (**C**) HCEC cells were treated with vehicle (Ctrl) or TNFα (20 ng/mL) for 4 h or for 4 d. The gene-specific EBI3 DNA methylation was analyzed via direct bisulfite sequencing. Indicated CpG positions refer to the transcription start site. U unmethylated, M methylated.

Since TNFα induced changes in *DNMT*- and *TET*-mRNA levels, we next analyzed whether TNFα affected global DNA methylation and hydroxymethylation in HCEC. The levels of 5′-methyl-2′-deoxycytidine (5-mdC) and 5′-hydroxymethyl-2′-deoxycytidine (5-hmdC) relative to dC were measured via LC-MS/MS. As expected, stimulation of HCEC with DAC resulted in a significant decrease in 5-mdC/dC-levels (3.1%) as compared to the untreated control (5.4%), while TNFα had no impact on global DNA demethylation when applied alone or in combination with DAC (Figure 2A). Neither DAC nor TNFα induced a significant effect on global HCEC hydroxymethylation (Figure 2B).

Having demonstrated that TNFα did not affect global DNA demethylation, we aimed to investigate whether the TNFα-induced reduction of *DNMT* expression and increase of *TET3* expression resulted in a gene-specific DNA demethylation. Methylation analysis of five cytosine-guanine dinucleotides (CpGs) upstream of the *EBI3* TSS revealed no methylation in vehicle-treated HCEC (controls, Figure 2C). By contrast, in the control HCEC, the CpGs +47, +107, +118, and +131 downstream of the TSS were all methylated, while the CpG at +165 was not. Interestingly, upon inflammatory stimulation with TNFα for 4 h or 4 d, the CpG at +107 became unmethylated (Figure 2C). Thus, it appeared that TNFα induced *EBI3* gene-specific DNA demethylation, perhaps as a result of reduced *DNMT* expression and/or increased *TET3* expression.

### 2.3. The Synergistic Increase in EBI3 by DNMTi and Inflammatory Stimulus Occurs via NFĸB and p38 MAPK

Since *EBI3* is an NFĸB target gene [24,25] and NFĸB subunits can be regulated by histone modifications and DNA methylation [26,27], we hypothesized that the observed synergistic effect of combined DAC and TNFα treatment on *EBI3* expression in HCEC occurs via NFĸB.

**Figure 3:**
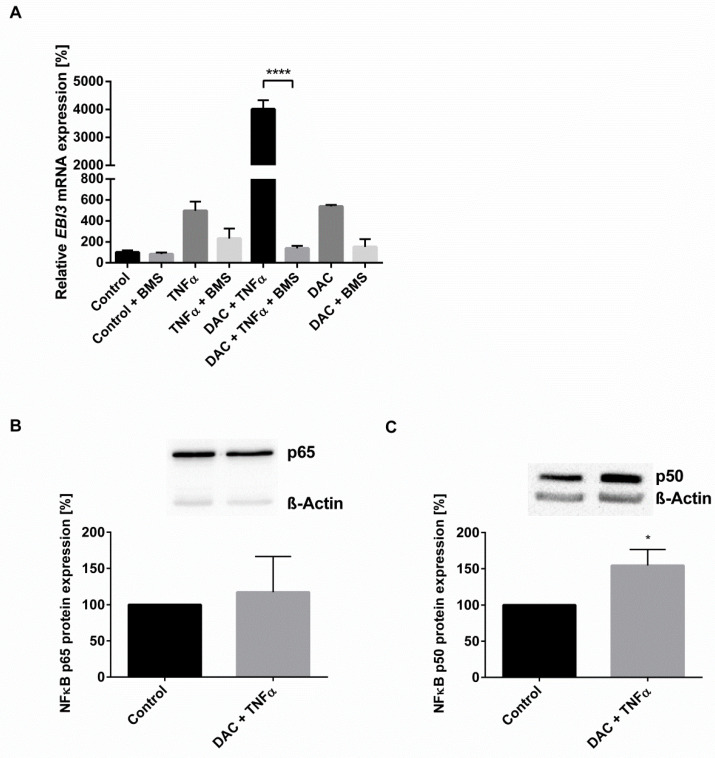
The synergistic effect of DAC and TNFα occurs via NFĸB. (**A**) HCEC cells were pretreated with BMS-345541 (5 µM) for 1 h before the stimulation with DAC (10 µM) or vehicle for 48 h with or without the addition of TNFα (20 ng/mL) for the final 24 h. The mRNA expression of EBI3 was measured with RT-qPCR. Glyceraldehyde-3-phosphate dehydrogenase (GAPDH) served as the reference gene. Data are shown as mean ± SEM from three independent experiments. Statistical analysis was performed using one-way ANOVA and Tukey’s post hoc test (**** *p* < 0.0001) (**B**,**C**) HCEC cells were stimulated with DAC (10 µM) for 48 h and TNFα (20 ng/mL) for the final 24 h. Cells stimulated with vehicle served as control. Whole-cell lysate proteins were separated by SDS-PAGE and the expressions of NFκB p65 (**B**) and NFĸB p50 (**C**) were detected by immunoblotting. ß-Actin served as loading control. Data are shown as mean ± SEM from three independent experiments. Statistical analysis was performed using the unpaired Student t test (* *p* < 0.05).

Inhibition of the NFκB pathway using the IκB kinase inhibitor BMS-345541 clearly impeded the DAC-stimulated *EBI3* induction in HCEC, with or without TNFα treatment. Addition of BMS-345541 (5 µM) reduced TNFα-mediated and DAC-induced *EBI3* expression by ~53% and ~72%, respectively (Figure 3A). Similarly, BMS-345541 caused a dose-dependent decrease in the *EBI3* induction by combined DAC and TNFα stimulation (Supplementary Figure S4A). A concentration of 5 µM BMS-345541 completely abolished the effect (Figure 3A). The applied doses of BMS-345541 were proven to be neither cytotoxic nor proliferative by means of MTT assays (Supplementary Figure S4B).

Moreover, protein expressions of NFĸB subunits in stimulated HCEC cells were investigated. Although treatment with DAC and TNFα did not influence the NFκB/p65 subunit (Figure 3B), it did result in a 54% increase to NFĸB/p50 protein expression as compared to the vehicle control (Figure 3C). This suggests NFκB/p50 signaling plays a key role in the mutually reinforcing effects caused by TNFα and DAC stimulation on *EBI3* expression.

**Figure 4:**
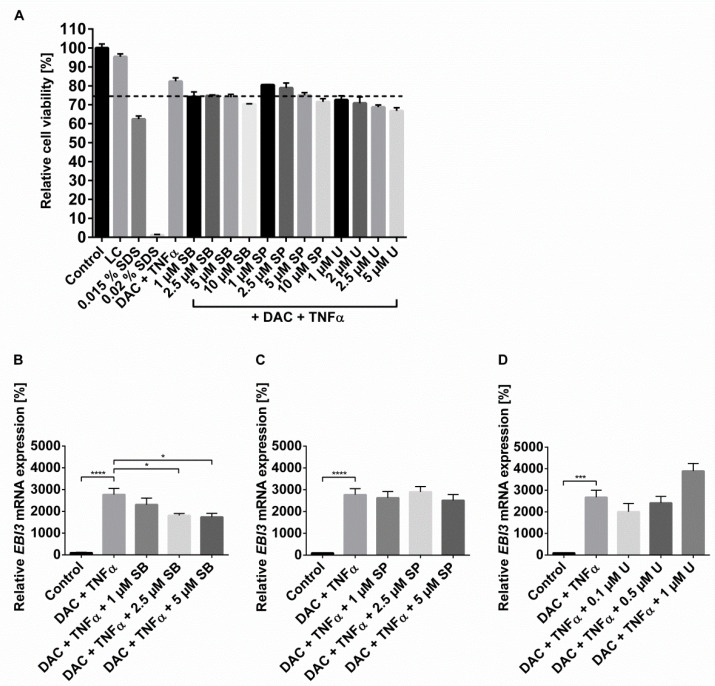
Inhibition of MAPKs influences the synergistic induction of *EBI3* levels by DAC and TNFα. (**A**) HCEC cells were treated with the indicated concentrations of p38 MAPKi SB203580, JNKi SP600125, ERKi U0126, DAC (10 µM), and TNFα (20 ng/mL) for 48 h. TNFα was added for the final 24 h. Cell viability was determined via MTT assay. The data are expressed as percentage of the untreated control and are shown as mean ± SEM from three independent experiments. (**B**–**D**) HCEC cells were pretreated with the indicated concentrations of p38 MAPKi SB203580 (**B**), JNKi SP600125 (**C**), and ERKi U0126 (**D**) for 1 h before the stimulation with DAC (10 µM) or vehicle for 48 h with the addition of TNFα (20 ng/mL) for the final 24 h. The mRNA expression of *EBI3* was measured with RT-qPCR. *HMBS* served as the reference gene. Data are shown as mean ± SEM from three independent experiments. The differences in *EBI3* mRNA level compared to the DAC and TNFα stimulation were statistically analyzed by one-way ANOVA and Tukey’s post hoc test (* *p* < 0.05; *** *p* < 0.001; **** *p* < 0.0001).

Further important signaling pathways induced by TNFα are the three groups of mitogen-activated protein kinases (MAPK) p38 MAPK, the cJun NH_2_–terminal kinases (JNK) and extracellular-signal-regulated kinases (ERK). In order to examine if MAPK also play a role in DAC+TNFα-induced *EBI3* induction, HCEC were pretreated with noncytotoxic concentrations of p38 MAPK inhibitor (MAPKi) SB203580, JNK inhibitor (JNKi) SP600125, and ERK inhibitor (ERKi) U0126 with subsequent stimulation with DAC and TNFα (Figure 4A). Inhibition of p38 MAPK reduced the synergistic effect of DAC+TNFα in a concentration-dependent manner with a significant decrease of 37% (5 µM SB203580) compared to DAC+TNFα alone (Figure 4B), while the effects of 5 µM SB203580 on cells treated separately with TNFα or DAC were not significant (Supplementary Figure S5). The treatments with JNKi SP600125 (Figure 4C) or with the ERKi U0126 (Figure 4D) did not significantly alter the synergistic effect of DAC+TNFα.

### 2.4. DNMTi Enhance EBI3 Dimer Formation and Secretion under Inflammatory Conditions

**Figure 5:**
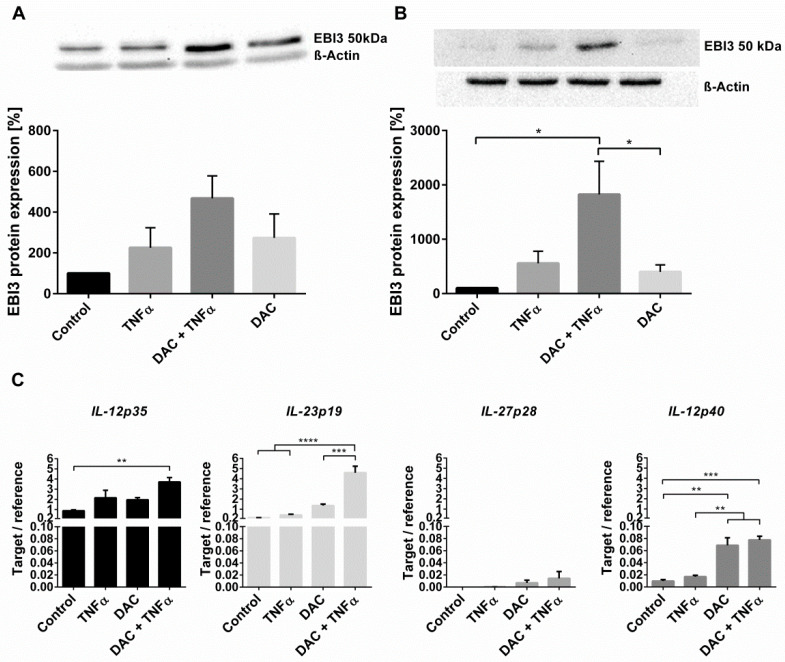
DAC and TNFα induce EBI3 protein expression and secretion. (**A**,**B**) HCEC were stimulated with DAC (10 µM) for 72 h with or without the addition of TNFα (20 ng/mL) for the final 24 h. The expression of EBI3 in HCEC cell lysates was determined via immunoblotting. Results were normalized to ß-Actin (**A**). The expression of EBI3 in HCEC cell culture media was analyzed via immunoblotting after trichloroacetic acid precipitation. To visualize that the supernatant came from the same number of attached cells, the same volumes of whole cell lysates were analyzed via immunoblotting with anti-β-Actin antibody (**B**). The graphs show the mean ± SEM from four independent experiments. Differences in EBI3 protein levels were analyzed using one-way ANOVA and Tukey’s post hoc test (* *p* < 0.05). (**C**) HCEC cells were treated with DAC (10 µM) for 48 h with or without the addition of TNFα (20 ng/mL) for the final 24 h. The mRNA expression of IL-12-cytokine subunits was determined via RT-qPCR. Results were normalized to HMBS. Data are shown as mean ± SEM from three independent experiments. Statistical analysis was performed using one-way ANOVA and Tukey’s post hoc test (** *p* < 0.01; *** *p* < 0.001; **** *p* < 0.0001).

In order to confirm the *EBI3* mRNA results, the influence of DAC and TNFα treatment on the formation and secretion of EBI3 proteins was determined via immunoblotting. Combinatorial stimulation of HCEC resulted in the highest observed increases to EBI3 levels in both cell lysate (Figure 5A) and cultured media (Figure 5B) (4.6- and 18.2-fold, respectively, compared to control). EBI3 (25.4 and 23.3 kDa with and without its signaling peptide, respectively) is a subunit of IL-35 when partnered with IL-12p35 (24.9 and 22.5 kDa) and of IL-39 when partnered with IL-23p19 (20.7 and 18.7 kDa; UniProt database). Therefore, the detection of protein bands ~50 kDa indicates dimer formation.

To investigate which of the IL-12 family dimeric cytokines was formed, mRNA levels of the possible EBI3 binding partners were determined. The subunits *IL-12p35* and *IL-23p19* were constitutively expressed in HCEC and their mRNA levels increased by 4.3- and 27.7-fold, respectively, upon combined DAC and TNFα stimulation, meaning enhanced formation of IL-35 and IL-39 were both possible (Figure 5C). By contrast, *IL-27p28* was barely expressed, making formation of IL-27 unlikely. Additionally, the mRNA level of *IL-12p40*, a further member of the IL-12 cytokine family was determined. This subunit is unable to bind EBI3 but could still compete for the EBI3-binding partners IL-12p35 or IL-23p19. DAC increased *IL-12p40* expression as compared to the vehicle control (about 7-fold). Subsequent treatment with TNFα had no further impact.

### 2.5. Combined DAC and TNFα Treatment Enhance IL-35 Production

**Figure 6:**
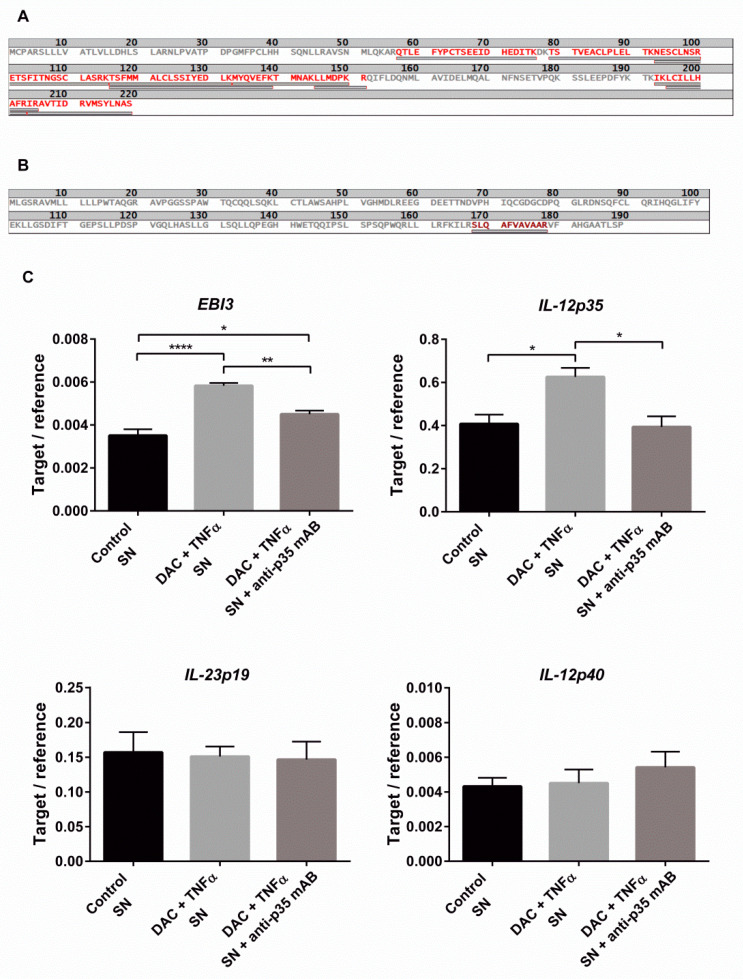
Indirect and functional proof of IL-35 cytokine production by HCEC. (**A**,**B**) HCEC cells were stimulated with DAC (10 µM) for 72 h and with TNFα (20 ng/mL) for the last 24 h. The whole-cell proteins were separated by SDS-PAGE and bands at a height of ~50 kDa were cut out. Following tryptic digestion, the resulting peptides were measured via MALDI-TOF-MS in the reflector mode operation over the mass range of m/z 500–5000. Detected peptide sequences of the IL-12p35 protein (**A**) and of the IL-23p19 protein (**B**) from three independent experiments. (**C**) HCEC cells were stimulated with DAC (10 µM) for 72 h and with TNFα (20 ng/mL) for the last 24 h. The cells were washed with PBS and cultured with fresh, untreated media for another 24 h to facilitate IL-35 secretion. HCEC cells were stimulated with the supernatant (SN) with or without the addition of neutralizing IL-35 antibody anti-p35 mAB (clone27537) (25 µg/mL) for 16 h. The mRNA expressions of EBI3, IL-12p35, IL-27p28, IL-23p19, and IL-12p40 were measured with RT-qPCR using HMBS as the reference gene. Data are shown as mean ± SEM from three independent experiments. Statistical analysis was performed using one-way ANOVA and Tukey’s post hoc test (* *p* < 0.05; ** *p* < 0.01; **** *p* < 0.0001).

Due to a current lack of specific antibodies against the complete IL-35 and IL-39 cytokines, their direct detection was not possible [14,28]. Instead, two indirect methods were used to investigate which dimer was formed. After stimulating HCEC with DAC and TNFα, the extracted proteins of the cell lysate were separated by SDS-PAGE, after which an in-gel tryptic digest was performed on an excised band found at ~50 kDa. The resulting peptides were analyzed via MALDI-TOF/MS. Ten peptides of the IL-12p35 protein-with a sequence coverage of over 50% were detected (Figure 6A), while only one short sequence section of IL-23p19 (5.8% sequence coverage) was found (Figure 6B), suggesting the predominant formation of IL-35 instead of IL-39. Further evidence of the presence of IL-12p35 was given by performing a targeted HPLC-ESI-MS/MS peptides analysis in the cell lysate. Here, too, only IL-12p35 was reliably detected, as documented in Supplementary Table S1.

A previous study has shown that IL-35 is able to stimulate the mRNA expression of their own subunits [29,30]. HCEC cells, exhibiting a basal expression of the IL-35 receptor subunits, *IL-12Rβ2* and *gp130* (Supplementary Figure S6), were stimulated with DAC and TNFα to enhance the *EBI3* and *IL-12p35* expressions. After 72 hours, the cells were washed with PBS to remove the stimulants and incubated with fresh media for 24 h to allow the accumulation of secreted IL-35. Subsequently, the cultured supernatants were collected and applied to untreated HCEC for 16 hours with or without the addition of the IL-35-neutralizing antibody. RT-qPCR of these revealed a significant increase in *EBI3* and an enhancement of *IL-12p35* mRNA levels as compared to the vehicle control (1.7- and 1.5-fold, respectively) (Figure 6C), demonstrating the formation of functional IL-35. Neutralization of IL-35 by anti-p35 mAB blocked the induction of *EBI3* and *IL-12p35*, verifying the production of IL-35 proteins by DAC+TNFα. The expression levels of *IL-23p19* and *IL-12p40* were not affected, while the mRNA of *IL-27p28* was undetectable. All experiments clearly indicate the formation of IL-35.

### 2.6. The Epigenetic Mechanisms Histone Acetylation and DNA Methylation Are Mutually Reinforcing in the Regulation of EBI3

**Figure 7:**
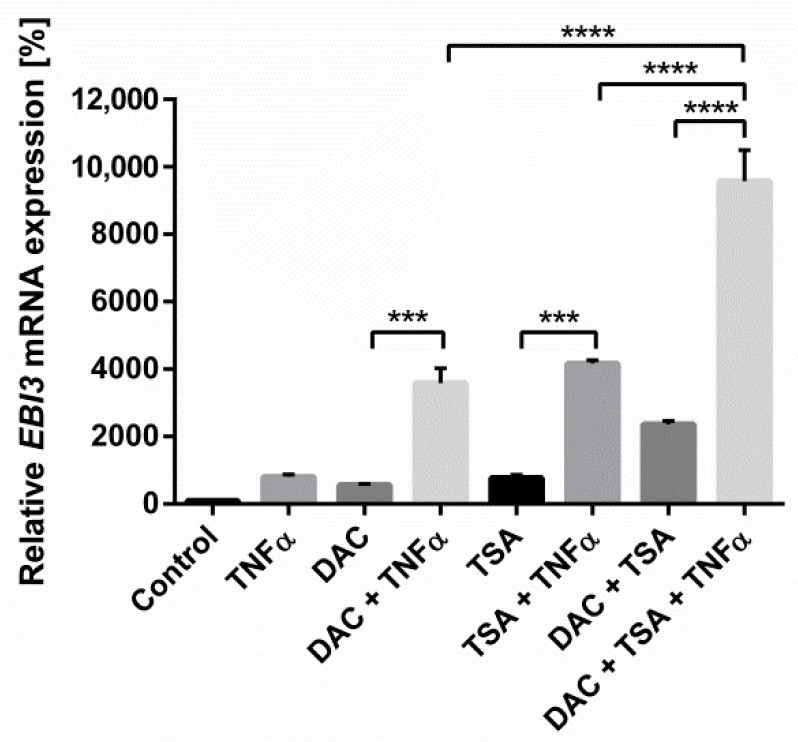
The combinatorial stimulation with DNMTi, histone deacetylase inhibitor (HDACi) and TNFα further enhances the increase in EBI3 mRNA expression. HCEC cells were stimulated with DAC (5 µM) and/or Trichostatin A (TSA) (0.1 µM) for 48 h and/or TNFα (20 ng/mL) for the last 24 h. The mRNA expression of EBI3 was measured with RT-qPCR with HMBS as the reference gene. Data are shown as mean ± SEM from three independent experiments. Statistical analysis was performed using one-way ANOVA and Tukey’s post hoc test (*** *p* <0.001; **** *p* <0.0001).

In our previous study, we revealed the influence of histone modification on *EBI3* expression in HCEC [19]. Because of the known dynamic link between histone acetylation and DNA methylation, we aimed to determine the effect of a combined HCEC treatment with HDACi, DNMTi, and TNFα. Individual stimulation with Trichostatin A (TSA), DAC, or TNFα resulted in 7.8-, 5.7-, and 8.1-fold increases in *EBI3*, respectively (Figure 7). Combined stimulation of TSA and TNFα upregulated *EBI3* by 41.7-fold, comparable to DAC/TNFα treatment. Parallel stimulation with DAC and TSA led to a 23.6-fold upregulation compared to the vehicle control. Finally, simultaneous treatment with all stimulants resulted in a 96-fold increase, indicating a mechanism where DNA methylation, histone acetylation, and inflammatory processes work cooperatively to up-regulate *EBI3*.

## 3. Discussion

UC is a multifactorial disease with unclear etiology, where imbalances within the immune system as well as the gut microbiome that may lead to epithelial barrier dysfunction are thought to play a role [31,32]. Dysbiosis alters bacterial production of bioactive substances such as folate, butyrate, or acetate, which are involved in epigenetic processes such as DNA methylation or histone modifications [33]. These epigenetic mechanisms may interfere in the development of IBD as well as regulation of cytokine levels. Recently, histone acetylation was shown to regulate the expression of *EBI3*, as part of the anti-inflammatory IL-12 cytokine family member IL-35 (EBI3/IL-12p35), in noncancerous cells generated from healthy human colon epithelium, as well as in a murine model of DSS-induced chronic colitis. Under inflammatory conditions, *EBI3* was induced by the HDACi TSA and SAHA [19]. A pivotal role for immune homeostasis has been described for this IL-12 cytokine family member. IL-35 induces the expression of additional immune-regulatory cytokines, such as IL-10 and TGF-β, that suppress immune cell inflammatory responses [34]. Notably, IL-35 was shown to play an anti-inflammatory role in UC [35,36]. *EBI3* expression is controlled in an inflammation-dependent manner through toll like receptor signaling and NFκB, as shown in dendritic cells [37]. Since histone modifications are often associated with further epigenetic processes [20], the potential impact of DNMTi on *EBI3* expression in inflamed cells was explored.

We show that DNA methylation regulates *EBI3* expression in human colon epithelial cells alongside histone acetylation. At first, *EBI3* mRNA expression was increased by demethylating conditions, as seen upon treatment of HCEC with the DNMTi DAC. Here, *EBI3* induction was comparable to that presented upon inflammatory challenge with TNFα. DAC also induced *EBI3* in other human cell types, including HUVEC, keratinocytes, and Raji cells, indicating this effect is not only limited to intestinal cells. Dual treatment of DAC and TNFα drove an apparently synergistic *EBI3* upregulation in HCEC, with similar impacts also noted in other cell types, including endothelial and epidermal cells. Only in the malignant B-cell line Raji was no TNFα-induced response observed, likely a result of their lack of TNFα membrane receptors [38]. As is well known, TNFα binding positively regulates NFκB signaling, a pathway of which *EBI3* expression is a target [21,25]. An *EBI3*-inducing effect in HCEC was also observed with the second demethylating substance used, AZA, thus confirming the epigenetic regulation mechanism. The lesser extent of the *EBI3* induction by AZA could be due to the fact that AZA is mainly incorporated into RNA, thereby leading to apoptosis. Only 10–20% of AZA are incorporated into DNA to substitute for cytosine. DAC, on the other hand, is completely incorporated into DNA [39].

TNFα has been reported to act on epigenetic mediators, e.g., by influencing the expression of DNMT [40], HDAC [41], and TET enzymes [42], or via the recruitment of mediators to specific gene regions. For example, TNFα is able to recruit either DNMT1, via phosphorylation of RelA/p65 [43], or DNMT3b together with the histone methyltransferase enhancer of zeste homolog 2 [44] to specific gene promoters. In HCEC cells, TNFα treatment caused an immediate decrease in *DNMT3a* and *DNMT3b* expression, while reduction in *DNMT1* levels was relatively delayed. With regards to demethylating enzymes, TNFα treatment decreased *TET1*, while *TET2* mRNA levels were unaffected. By contrast, *TET3* expression initially increased following the first 2 h of TNFα treatment, falling to basal values over the next 14 h. Since basal expression of *DNMT1* and *TET3* are much higher than of their other isoforms, these can be considered of particular relevance to HCEC. The downregulation of *TET1* by TNFα has already been described in several publications. Morisawa et al. detected downregulation of a TNFα-target gene by reduction of TET1 in fibroblasts [42]. Haseeb et al. investigated IL-1ß and TNFα-treated chondrocytes and found a decrease in hydroxymethylation near the transcription start sites of several specific genes known to be upregulated by TNFα and IL-1ß [45]. As a possible mechanism, they suggest that the activation of NFκB by TNFα led to the downregulation of *TET1* since the application of an NFκB inhibitor abolished the TNFα-mediated reduction.

In the present study, global methylation analysis demonstrated decreased 5′-mdC levels in HCEC on DAC treatment, but not TNFα stimulation. However, gene-specific methylation analysis revealed TNFα treatment could in fact induce local hypomethylation at the specific CpG +107 bp downstream of the *EBI3* TSS, perhaps explaining its inflammation-induced expression. Notably, the fast increase to the demethylating *TET3* levels alongside reductions to *DNMTs* after TNFα treatment might also play a role. To better understand the mechanism behind this effect, a complete methylation analysis of the promoter should be performed. As a relevant comparison, TNFα was reported to induce demethylation at a single CpG located 36 bp upstream of the matrix metalloproteinase 9 TSS, resulting in a slightly increased transcriptional activity [46].

Our results indicate that the synergistic effect of DAC and TNFα on *EBI3* expression is largely mediated by NFκB. Firstly, the IκB kinase inhibitor BMS-345541 reduced the *EBI3* expression induced by TNFα or DAC treatment and dose-dependently abolished the synergism upon their parallel application to HCEC. Secondly, increased protein expression of the NFκB subunit p50 (NFκB1) was seen on DAC and TNFα stimulation. NFκB, as downstream target of TNFα, can directly induce the transcription of *EBI3*. We show that TNFα could also improve the accessibility of *EBI3* to transcription factors such as NFκB by hypomethylation of the *EBI3* gene. However, it must be noted that the sequence environment of the *EBI3* CpG +107 bp is not an NFκB binding site. Instead, two upstream NFκB binding sites in the *EBI3* promoter are reportedly important to *EBI3* regulation [25]. Since NFκB promotor can also be methylated, DAC could induce hypomethylation here and subconsequently stimulate NFκB expression, in turn leading to enhanced *EBI3* expression [26,47].

To prove if MAPK, as other important signaling pathways induced by TNFα, may play a role in DAC+TNFα-mediated *EBI3* induction in HCEC, we tested inhibitors for the three groups of MAPK, p38 MAPK, JNK, and ERK. Blocking of the JNK and ERK pathways did not alter the *EBI3* expression, whereas the pre-incubation with p38 MAPKi SB203580 resulted in a reduced *EBI3* mRNA induction in comparison to the combined stimulation with DAC+TNFα. However, the effect was not as potent as the IκB kinase inhibitor BMS-345541, which almost completely abolished the effect. As several studies have demonstrated that the expression of *EBI3* through various stimuli is regulated by NFκB and not via MAPK [37,48,49] and also because the link between p38 signaling and NFκB-mediated gene expression is well known [50,51,52], the results suggest that part of the NFκB effect is mediated via p38 MAPK.

DAC and TNFα treatment increased EBI3 protein levels in both HCEC cell lysates and cell culture supernatants, as would be expected of a secretory protein. Western blotting against EBI3 revealed protein bands of ~50 kDa, indicative of heterodimer formation with another cytokine subunit. Indeed, the possible EBI3 binding partners *IL-12p35* and *IL-23p19* are expressed in HCEC. Direct evidence of IL-35 (EBI3/IL-12p35) or IL-39 (EBI3/IL-23p19) formation is currently not possible due to the similar size of both heterodimers and a lack of specific antibodies that do not cross-react with the other subunits [14,28,53]. Instead, two indirect methods were employed.

Firstly, the dimeric 50 kDa protein product resolved by SDS-PAGE was excised, tryptically digested, and analyzed by MALDI-TOF-MS. While several peptides for the IL-12p35 protein were found, with a sequence coverage of ~50%, just one predicted IL-23p19 peptide was detected. These results were additionally confirmed via HPLC-ESI-MS/MS, which was able to identify several IL-12p35 peptide sequences but could not reliably detect IL-23p19. Therefore, the formation of IL-39 appears highly unlikely. In addition, there are a number of doubts present within the current literature as to whether functional IL-39 does in fact form in humans [54,55]. Interestingly, recent data from an animal experiment performed by our group also indicated the formation of IL-35 rather than of IL-39 [19].

Additional functional proof of IL-35 formation was provided by the work of Collison et al. [29], who investigated the IL-35-mediated conversion of conventional T cells into regulatory T cells, iTr35. The authors stimulated murine conventional T cells with IL-35 and detected an upregulation of the IL-35 subunits *Ebi3* and *IL-12p35* via the transcription factors signal transducer and activator of transcription 1 and 4 (STAT1, STAT4). Both receptor subunits, IL-12Rß2 and gp130, were required for this induction.

In the present study, HCEC that express *IL-12Rß2* and *gp130* were challenged with the cell media supernatants of separate DAC- and TNFα- or vehicle-stimulated HCEC cells. Examination of mRNA levels revealed an upregulation of *EBI3* and *IL-12p35* in the treated cells, an effect abolished by the neutralizing IL-35 antibody, confirming the treatment-induced formation of functional IL-35 protein.

We previously demonstrated that *EBI3* was also upregulated in HCEC following treatment with the HDACi TSA under inflammatory conditions. Parallel incubation with DAC and TSA resulted in over-additive upregulation of *EBI3* mRNA expression. Remarkably, addition of the pro-inflammatory cytokine TNFα to this resulted in even greater synergistic increase to *EBI3* expression. Early findings by Cameron et al. demonstrated the cooperation between histone modification and DNA methylation and their synergism for gene reactivation [56]. The relationship between these two types of modification seems to be mediated to some extent by 5-mdC binding proteins, such as Methyl-CpG binding protein 2 or Methyl-CpG binding domain protein 2, that are able to recruit HDAC to methylated parts of the genome [57,58]. Additionally, DNMTs can modulate patterns of histone acetylation or methylation by interaction with histone methyltransferases such as Suv39h1 or enhancer of zeste homolog 2 [59,60].

TSA inhibits class I and II HDACs but is not therapeutically used due to its pronounced toxicity. SAHA, another HDACi that inhibits class I and II HDACs, is already approved for the therapy of advanced refractory cutaneous T-cell lymphoma [61]. The beneficial effect of SAHA on colitis symptoms that we had observed was in agreement with investigations by others [62,63,64]. DAC is approved for the treatment of patients with myelodysplastic syndromes and of older patients with acute myeloid leukemia [65,66]. There are also studies showing an effect of DAC on inflammatory diseases such as experimental autoimmune encephalomyelitis [67], type 1 diabetes [68], Guillain-Barré syndrome [69], or collagen-induced arthritis [70]. To the best of our knowledge, there are no studies that use DAC for UC therapy. In some studies, the treatment of cancer cells with DNMTi in combination with HDACi has been shown to be more effective than the application of single substances [71,72,73,74,75]. Therefore, the combination of the DNMTi DAC and the HDACi SAHA in an inflammatory condition, such as inflammatory bowel disease, could be an effective therapeutic option that should be tested in murine DSS-induced chronic colitis in the future.

## 4. Materials and Methods

### 4.1. Cell Culture

HCEC, generated from healthy guts, were obtained from Nestlé Ltd. Research Centre (Lausanne, Switzerland) and were cultured in Dulbecco’s Modified Eagle’s Medium with 2 mM L-glutamine supplemented with 9.11 µL/mL sodium pyruvate, 4.9 × 10^−3^ µL/mL phosphoethanolamine, 4.9 × 10^−3^ µL/mL ethanolamine, 3 mg/mL bovine serum albumin, 10% fetal bovine serum, 45 IU/mL penicillin, and 45 IU/mL streptomycin. Human umbilical vein endothelial cells (HUVEC, provided by the Department of Biology, Chemistry, and Pharmacy, Freie Universität Berlin, Germany) were cultured with Endothelial Cell Growth Medium-2 BulletKit^TM^ (Lonza Group AG, Basel, Switzerland). Human primary keratinocytes, isolated from foreskin, were cultured with Keratinocyte Growth Medium-2 BulletKit^TM^ (Lonza Group AG, Basel, Switzerland). Raji, B-cells derived from Burkitt’s lymphoma, were grown with Roswell Park Memorial Institute 1640 medium supplemented with 10% fetal bovine serum, 45 IU/mL penicillin, and 45 IU/mL streptomycin.

Cultivation of cells was carried out in a humidified incubator at 37 °C with 5% CO_2_ in O_2_ until 90–95% confluency before subcultivation. For an experiment, 1 × 10^6^ cells were seeded on 150 mm diameter dishes (TPP Techno Plastic Products AG, Trasadingen, Switzerland). When cells were in the exponential growth phase, they were treated with 10 µM DAC (Sigma Aldrich, Steinheim, Germany) or vehicle for 48 h, unless otherwise indicated. For the last 24 h of the incubation, 20 ng/mL TNFα (Miltenyi Biotec GmbH, Bergisch Gladbach, Germany) or vehicle was added. For long-term stimulation (4 d) with TNFα, the stimulant was freshly added every 24 h. In some experiments, cells were treated with 0.1 µM 5-azacytidine (AZA); 3, 4, or 5 µM BMS-345541 (Sigma Aldrich, Steinheim, Germany); 1, 2.5, or 5 µM SB203580 (SB); 1, 2.5, or 5 µM SP600125 (SP); 0.1, 0.5, or 1 µM U0126 (U) (Cayman Chemical, Ann Arbor, MI, U.S.A), or 0.1 µM Trichostatin A (Sigma-Aldrich, Steinheim, Germany) for 48 h. TNFα was dissolved in 0.1% bovine serum albumin in PBS; all other substances in dimethyl sulfoxide with a maximum final dimethyl sulfoxide concentration of 0.1% in the reaction mixture. Before use, the cells were tested to rule out mycoplasma contamination.

### 4.2. Analysis of Gene Expression

Cells were stimulated as indicated. Isolation of total RNA was performed with the High Pure RNA Isolation Kit (Roche, Mannheim, Germany). The RNA concentration and purity were measured using the NanoVue^TM^ Plus UV-Vis spectrophotometer (GE Healthcare, Berlin, Germany). RNA with a ratio 2.0 of absorbance at 260/280 nm was reverse-transcribed with the RevertAid reverse transcriptase, according to the manufacturer’s instructions (Thermo Fisher, Darmstadt, Germany). Quantitative reverse transcription-PCR (RT-qPCR) was conducted with Maxima SYBR Green qPCR Mix (ThermoFisher, Darmstadt, Germany) and a LightCycler 480 II Real-Time PCR system (Roche, Mannheim, Germany). The ΔΔC_T_ method was used for relative quantification with *hydroxymethylbilane synthase* (*HMBS*) or *glyceraldehyde-3-phosphate dehydrogenase* (*GAPDH*) as reference genes [76]. Suitability of the chosen reference gene was rechecked for every experimental condition to ensure stable expression without being influenced by test substances. The oligonucleotide primers used are listed in Table 1. Primer specificity for positive controls was assessed via RT-qPCR melt profile analysis and the formation of correctly sized PCR products on ethidium bromide-stained agarose gels following electrophoresis. PCR conditions were as follows: 42 cycles of 15 s at 95 °C, 15 s at annealing temperature (60 °C for *h-IL12p40* and *h-IL-23p19*, and 58 °C for the other genes), and 15 s at 72 °C. The samples were measured in duplicates of at least three independent experiments.

### 4.3. Analysis of Genome-Wide DNA Methylation and Hydroxymethylation

HCEC cells were treated with DAC (10 µM) or vehicle for 48 h. For the last 24 h of incubation TNFα (20 ng/mL) or vehicle was also added. Genomic DNA was isolated with the QIAamp DNA Mini Kit (Qiagen, Hilden, Germany). Global changes to 5-mdC/dC and 5-hmdC/dC levels were, after two-step hydrolysis of 20 µg genomic DNA to 2′-deoxynucleosides, analyzed via liquid chromatography tandem-mass spectrometry (LC-MS/MS), as described previously [77].

### 4.4. Analysis of Gene-Specific DNA Methylation

To analyze gene-specific DNA methylation, HCEC cells were stimulated with/without TNFα for 4 h and for 4 d. For 4 d incubation times, fresh TNFα was added daily. Genomic DNA was isolated from cell pellets with the QIAamp® DNA Mini Kit (Qiagen, Hilden, Germany). Bisulfite conversion of unmethylated cytosines into uracil was performed with the EZ DNA Methylation^TM^ Kit (Zymo Research, Irvine, U.S.A), according to the manufacturer’s specifications. Detection of methylated cytosine–guanine dinucleotides (CpGs) surrounding the *EBI3* gene transcription start site (TSS) was determined by means of nested (upstream of TSS) or seminested (downstream) PCR amplification of bisulfite-converted DNA followed by direct Sanger sequencing (Eurofins Genomics and GATC, Konstanz, Germany). PCR conditions were as follows: 40 cycles of 45 s at 95 °C, 20 s annealing, 30 s at 72 °C. Annealing temperatures were 50 °C each for the first up- and downstream PCR, 48 °C for the second upstream PCR, and 55 °C for the second downstream PCR. Oligonucleotide primers used are listed in Table 2.

### 4.5. Immunoblotting

HCEC cells were treated for 72 h with DAC (10 µM) or vehicle, with/without the addition of TNFα (20 ng/mL) for the last 24 h. Protein from whole-cell lysates was boiled in SDS sample buffer, separated by 12% SDS-polyacrylamide gel electrophoresis (SDS-PAGE) and blotted onto PVDF membranes. The membranes were first incubated with 5% nonfat dry milk in Tris-buffered saline/Tween^®^20 for 1 h at room temperature, then with primary antibodies overnight at 4 °C: anti-NFκB p65 (F-6), sc-8008 and anti-NFκB p50 (E-10), sc-8414 (Santa Cruz Biotechnology, Dallas, TX, USA); anti-EBI3 (EPR5747), ab124694 (Abcam, Cambridge, UK). Membranes were subsequently washed with Tris-buffered saline/Tween^®^20 and incubated with a secondary antibody—anti-rabbit IgG HRP linked antibody (#7074, Cell Signaling Technology, Frankfurt, Germany)—for 1 h at room temperature. After detection with Clarity Western ECL Substrate, according to the manufacturer’s protocol using a ChemiDoc XRS+ system (Bio-Rad Laboratories, Munich, Germany), the membranes were incubated with anti-ß-Actin (ab8226, Abcam, Cambridge, UK) as loading control for 1 h at room temperature. Band detection was again conducted as described above.

### 4.6. Trichloroacetic Acid Precipitation

For the detection of EBI3 in cell culture supernatants, HCEC cells were stimulated for 72 h with DAC (10 µM) or vehicle. For the last 24 h of incubation TNFα (20 ng/mL) was added. Cultured cell media were collected and incubated with 10% trichloroacetic acid for 15 min on ice. After centrifugation (12,000× *g*, 5 min, 4 °C), the resulting pellets were washed with ice-cold acetone and resuspended with PBS. The samples were neutralized with 1 M Tris, boiled in SDS sample buffer, and analyzed via immunoblotting. In order to prove that the secreted proteins came from the same number of attached cells, the corresponding cells were lysed in RIPA buffer and the total protein content was determined via Bradford assay [78]. For visualization, the same volumes of cell lysates were analyzed via immunoblotting against β-Actin (ab8226 Abcam, Cambridge, UK), as described above.

### 4.7. Matrix-Assisted Laser Desorption/Ionization Time-of-Flight Mass Spectrometry (MALDI-TOF-MS)

HCEC cells were stimulated for 72 h with DAC (10 µM) or vehicle with/without the addition of TNFα (20 ng/mL) for the last 24 h. Proteins of whole-cell lysates were separated by SDS-PAGE. After incubation with a colloidal Coomassie staining solution, bands found at 50 kDa were excised, and an In-Gel Tryptic Digestion Kit (Thermo Scientific^TM^ PIERCE^TM^) was applied. The resulting digest was mixed 1:1 with the matrix solution (α-cyano-4-hydroxy cinnamic acid, HCCA) and 1 µl of this mixture was applied onto a steel plate for MALDI-TOF-MS analysis (Bruker Daltonic GmbH, Bremen, Germany). TOF-MS measurements were performed in the reflector mode operation over the mass range of m/z 500–5000. Further details for methodical parameters are given in the here cited literature [79].

### 4.8. Targeted HPLC-ESI-MS/MS Peptides Analysis for IL-12p35

Tryptic digested proteins of whole cell lysates (see details in Supplementary Table S1) from HCEC cells stimulated for 72 h with vehicle or DAC (10 µM) with the addition of TNFα (20 ng/mL) for the last 24 h were analyzed using an Agilent Infinity 1260 system with binary pump, multicolumn thermostat, and auto sampler equipped with an Agilent G6470A Series Triple Quad LC/MS (both from Agilent Technologies Sales & Services GmbH & Co.KG, Waldbronn, Germany) coupled with an electrospray source operating in positive ionization mode. Peptides were separated using a Kinetex C8 analytical column (2.6 µm, 100 A, 150 × 4.60 mm^2^; Phenomenex, Torrance, CA, USA) kept at a temperature of 30 °C. The mobile phase was composed of eluent A (0.1% formic acid) and eluent B (acetonitrile), at a flow rate of 0.5 mL min^−1^.

The following mobile phase gradient conditions were applied: 100% A from 0 to 2 min, 100–50% A from 2 to 18 min, 50–5% A from 18 to 19 min, 5% A from 18 to 22 min, 5–99% A from 22 to 23 min, and 99% A from 23 to 28 min. The column equilibration time between each run was 4 min at 100% A. A volume of 20 µL of the sample was injected. The desolvation gas temperature in the ionization source was set at 275 °C. The gas flow was 11 L min^−1^, the nebulizer pressure was 35 psi, and the fragmentor voltage was set at 130. Detection was performed in the selected reaction monitoring mode, in which a specific transition was monitored at a specific time according to the retention time of the peptides.

For the application of the selected reaction monitoring method, retention times and collision energies for the transitions were generated using the MacCoss Lab Skyline software (University of Washington, Seattle, WA, USA). Up to maximum five transitions for each peptide were selected. Thereafter, the relative abundance of each peptide was measured under consideration of the total area of all the transitions analyzed. The supplementary information documents the conditions for the peptides selected, HPLC and MS conditions applied, and the response recorded (Supplementary Table S1).

### 4.9. Functional Proof of IL-35 Production

HCEC cells were stimulated with DAC (10 µM) for 72 h with the addition of TNFα (20 ng/mL) for the last 24 h. Control cells were treated with vehicles. The cells were washed with PBS to remove the stimulants and then cultured in fresh media for another 24 h to facilitate IL-35 production and secretion. The supernatants of these cells were collected, centrifuged to remove possible cell contamination, and applied to another set of untreated HCEC cells for 16 h with or without the addition of the IL-35-neutralizing antibody (anti-p35 mAB, MAB1570, R&D Systems, Minneapolis, U.S.A) (25 µg/mL) [80]. The mRNA expression levels of *EBI3, IL-12p35, IL-27p28, IL-23p19*, and *IL-12p40* in these new HCEC were measured with RT-qPCR.

### 4.10. MTT Assay

To determine possible cytotoxic effects induced by BMS-345541, SB203580, SP600125, U0126, DAC, and TNFα, a 3-(4,5-dimethylthiazol-2-yl)-2,5-diphenyltetrazolium bromide (MTT, Sigma-Aldrich, Steinheim, Germany) reduction assay (MTT assay) was used [81]. Eight thousand HCEC cells per well were seeded into 96-well plates (TPP, Trasadingen, Switzerland). The cells were treated for 48 h with different concentrations of the substances, beginning 24 h after seeding. For the parallel stimulation with DAC and TNFα, TNFα was added for the final 24 h. Positive controls were treated with 0.015 and 0.02% SDS. Untreated controls were incubated for the same period. After the incubation time, the cells were washed with PBS and treated with 100 μl MTT solution per well (0.5 mg/mL) for 4 h at 37 °C. Afterward, the supernatants were removed and 50 μl dimethyl sulfoxide was added. To dissolve the formazan salt, the plates were shaken at 300 rpm for 10 min at room temperature. The optical density at 540 nm was measured using a microplate reader (Tecan, Crailsheim, Germany). A cell viability <75% predicts cytotoxic effects.

### 4.11. Statistical Analysis

Data are presented as mean ± standard error of the mean (SEM). Statistical analysis was performed using the unpaired Student *t* test, one- and two-way-ANOVA with Tukey’s or Sidak’s post hoc test (* *p* < 0.05; ** *p* < 0.01; *** *p* < 0.001; **** *p* < 0.0001) with the software GraphPad Prism (GraphPad Software, Inc., La Jolla, USA).

## 5. Conclusions

In addition to histone modification, *EBI3* is epigenetically regulated by promoter methylation in human colon epithelial cells. Synergistic induction of *EBI3* by inflammatory and methylation-inhibiting agents involves NFĸB signaling. Since EBI3 seems to form the anti-inflammatory dimeric cytokine IL-35, which is able to restrict colitis symptoms, rather than the pro-inflammatory IL-39, increased *EBI3* expression may contribute to colitis improvement. Therefore, *EBI3*-inducing DNMTi alone or, especially, in combination with HDACi, such as SAHA, might represent a therapeutic option in inflammatory diseases like colitis to alleviate symptoms and prevent colitis-associated cancer development.

## Figures and Tables

**Table 1:** Oligonucleotide primer sequences and product sizes for RT-qPCR experiments.

Target Gene	Gene Accession Number	Sequence	Fragment Size (bp)
*h-HMBS*	NM_000190.3	fw: ACCAAGGAGCTTGAACATGC	143
		rv: GAAAGACAACAGCATCATGAG	
*h-GAPDH*	NM_002046.7	fw: TGATGACATCAAGAAGGTGG	244
		rv: TTACTCCTTGGAGGCCATGT	
*h-EBI3*	NM_005755.2	fw: ATTGCCACGTACAGGCTCGG	131
		rv: ACATTGAGCACGTAGGGAGC	
*h-IL-12p35*	NM_000882.3	fw: ACAGTGGAGGCCTGTTTACC rv: ACTCCCATTAGTTATGAAAGAGGTC	87
*h-IL-12Rß2*	NM_001258216.1	fw: TTGTCTGCAAGGAGAAGACACA rv: ACTTCACTGATGACCAGCGG	94
*h-gp130*	NM_002184.3	fw: GGAGTGAAGAAGCAAGTGGGA rv: AGGCAATGTCTTCCACACGA	128
*h-IL-27p28*	NM_145659.3	fw: CAGGCGACCTTGGCTGG rv: CAGGTGAGATTCCGCAAAGC	206
*h-IL-12p40*	NM_002187.2	fw: GCCCAGAGCAAGATGTGTCA rv: CACCATTTCTCCAGGGGCAT	150
*h-IL-23p19*	NM_016584.2	fw: AGGCAAAAAGATGCTGGGGA rv: TCCTTTGCAAGCAGAACTGAC	287
*h-DNMT1*	NM_001130823.1	fw: AGACTACGCGAGATTCGAGTC rv: TTGGTGGCTGAGTAGTAGAGG	171
*h-DNMT3a*	NM_175629.2	fw: CGCAAAGCCATCTACGAGGTC rv: GGGATTCTTCTCTTCTTCTGGTGG	198
*h-DNMT3b*	NM_006892.3	fw: AATGTGAATCCAGCCAGGAAAGGC rv: ACTGGATTACACTCCAGGAACCGT	191
*h-TET1*	NM_030625.2	fw: GCTGCTGTCAGGGAAATCAT rv: ACCATCACAGCAGTTGGACA	209
*h-TET2*	NM_001127208.2	fw: CCAATAGGACATGATCCAGG rv: TCTGGATGAGCTCTCTCAGG	232
*h-TET3*	NM_001287491.1	fw: TCGGAGACACCCTCTACCAG rv: CTTGCAGCCGTTGAAGTACA	179

Gene accession numbers refer to the GenBank^®^ sequence database provided by the National Center for Biotechnology Information (NCBI, USA). *HMBS*, hydroxymethylbilane synthase; *GAPDH*, glyceraldehyde-3-phosphate dehydrogenase; *EBI3*, Epstein–Barr virus-induced gene 3; *IL*, interleukin; *DNMT*, DNA methyltransferase; *TET*, ten-eleven translocation; *fw*, forward; *rv*, reverse; *bp*, base pairs.

**Table 2:** Oligonucleotide primers for *EBI3* gene-specific methylation analysis and product sizes.

Reaction	Primer Sequence	Fragment Size (bp)
Nested PCR Upstream 1	GTTGTGTTGGGAAAAGTTAGTAGGTT	357
	AACAACTAACCCAAAAAACAAAAAC	
Upstream 2	TTAGTGAGTTAGATTTGAAGGAAGT	183
	AAAAACAAAAACCAAAAAAAACTAC	
Seminested PCR Downstream 1	GTTTTTGTTTTTTGGGTTAGTTGTT	230
	CACCCCCAATATAACACTCTACCTCT	
Downstream 2	GTTTTTGTTTTTTGGGTTAGTTGTT	211
	TACCTCTCTATACCTCAATTCCTCC	

## Data Availability

Not applicable.

## Supplementary Materials

The following are available online at https://www.mdpi.com/article/10.3390/ijms22105329/s1, Figure S1: DAC and TNFα induce a synergistic increase in *EBI3* mRNA expression, Figure S2: The demethylating agent 5-azacytidine (AZA) increases *EBI3* mRNA under inflammatory conditions in colon epithelium, Figure S3: Methylating and demethylating enzymes are inflammation-dependent, Figure S4: BMS-345541 dose-dependently inhibits the synergistic induction of *EBI3* levels by DAC and TNFα, Figure S5: The effects of p38 MAPKi on DAC- and TNFα-induced *EBI3* expression, Figure S6: Basal expression of IL-35 receptor subunits in HCEC, Table S1: HPLC-ESI-MS/MS analysis of IL12A (IL-12p35) and IL23A (IL-23p19).
