# Supplementary material for: Epigenetic DNA Methylation of EBI3 Modulates Human Interleukin-35 Formation via NFkB Signaling: A Promising Therapeutic Option in Ulcerative Colitis
Source: Int J Mol Sci. 2021 May 19;22(10):5329. doi: 10.3390/ijms22105329 (PMC8158689; doi:10.3390/ijms22105329)
Supplement: Supplementary file 1 [file ijms-22-05329-s001.zip › Wetzel et al supplements-IJMS.pdf]

Epigenetic DNA methylation of *EBI3* modulates human Interleukin-35 formation via NFkB signaling: a promising therapeutic option in ulcerative colitis

Alexandra Wetzel [1], Bettina Scholtka [1], Fabian Schumacher [2], Harshadrai Rawel [1], Birte Geisendörfer [1] and Burkhard Kleuser [2,*]

[1] Institute of Nutritional Science, University of Potsdam, Arthur-Scheunert-Allee 114-116, 14558 Nuthetal, Germany; alwetzel@uni-potsdam.de (A.W.); scholtka@uni-potsdam.de (B.S.); rawel@uni-potsdam.de (H.R.); geisendoerfer@uni-potsdam.de (B.G.)

[2] Institute of Pharmacy, Freie Universität Berlin, Königin-Luise-Str. 2+4, 14195 Berlin, Germany; fabian.schumacher@fu-berlin.de (F.S.); kleuser@zedat.fu-berlin.de (B.K.)

* Correspondence: kleuser@zedat.fu-berlin.de (B.K.)

Supplementary Figures

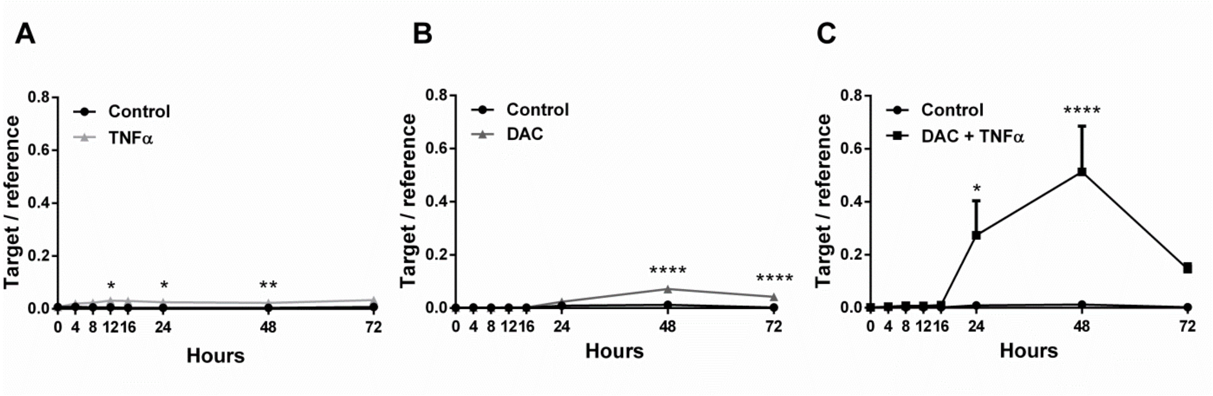

Supplementary Figure S1. DAC and TNFα induce a synergistic increase in *EBI3* mRNA expression. HCEC were stimulated for up to 72 h with TNFα (20 ng/ml) **(a)**, DAC (10 μM) **(b)** or both stimulants **(c)**. The *EBI3* mRNA expression was measured with RT-qPCR. Data were normalized to *HMBS* and compared to vehicle-stimulated cells by two-way ANOVA and Sidak's post hoc test. The graphs show the mean ± SEM from three independent experiments (*p < 0.05; **p < 0.01; ****p < 0.0001).

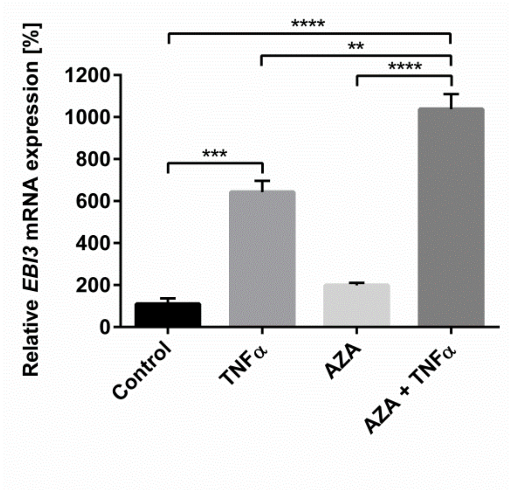

Supplementary Figure S2. The demethylating agent 5-azacytidine (AZA) increases *EBI3* mRNA under inflammatory conditions in colon epithelium. HCEC cells were treated with AZA (0.1 μM) for 48 h with or without the addition of TNFα (20 ng/ml) for the final 24 h. The relative quantification of *EBI3* mRNA expression was done with RT-qPCR. *HMBS* served as the reference gene. Data are shown as mean ± SEM from three independent experiments. Statistical analysis was performed using one-way ANOVA and Tukey's post hoc test (**p <0.01; ***p <0.001; ****p <0.0001).

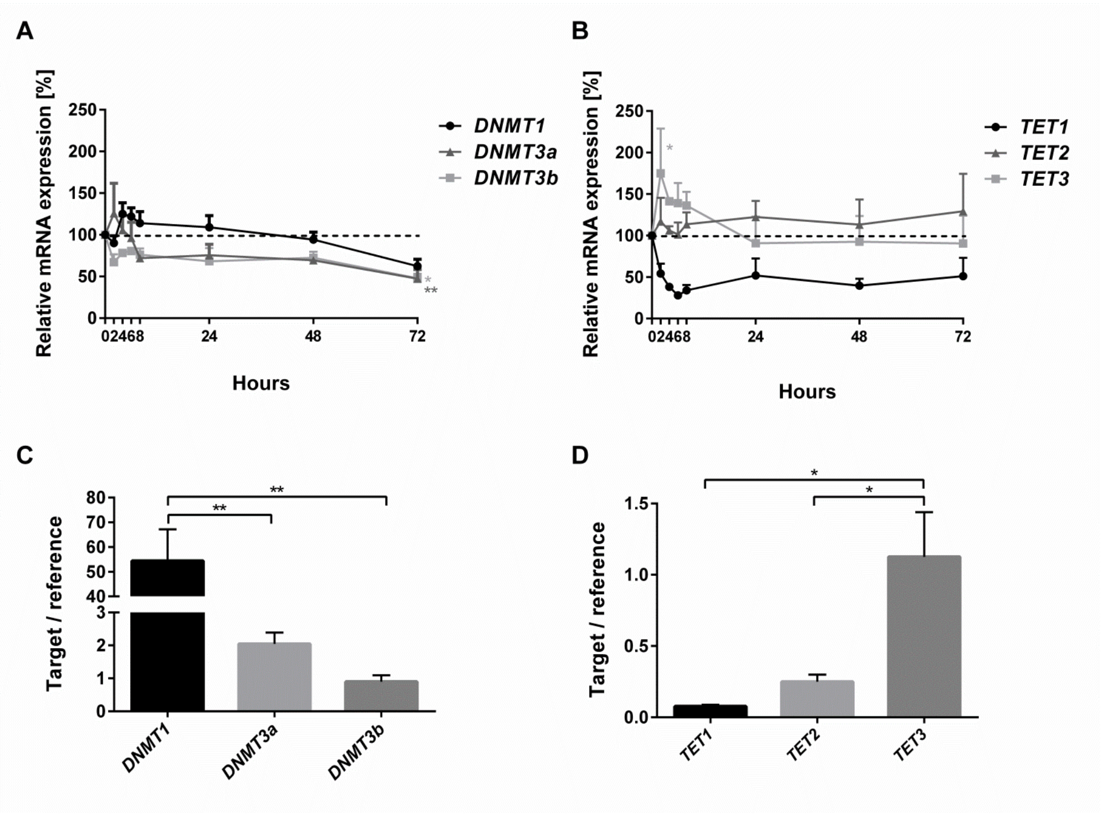

Supplementary Figure S3. Methylating and demethylating enzymes are inflammation-dependent. **(a, b)** HCEC cells were treated with TNFα (20 ng/ml) for the indicated periods. Vehicle controls were performed for every time point. The relative quantification of *DNMT1*, *DNMT3a* and *3b* **(a)**, and *TET1*, *TET2* and *TET3* **(b)** mRNA levels were determined by RT-qPCR and normalized against the reference gene *HMBS.* Data are shown as mean ± SEM from three independent experiments. Statistical analysis was performed using two-way ANOVA and Tukey's post hoc test (*p <0.05; **p <0.01). **(c, d)** The basal mRNA expressions of *DNMT1*, *DNMT3a* and *DNMT3b* **(c)** and of *TET1*, *TET2* and *TET3* **(d)** in HCEC were determined by RT-qPCR using *HMBS* as the reference gene. Data are shown as mean ± SEM from three independent experiments. Statistical analysis was performed using one-way ANOVA and Tukey's post hoc test (*p <0.05; **p <0.01).

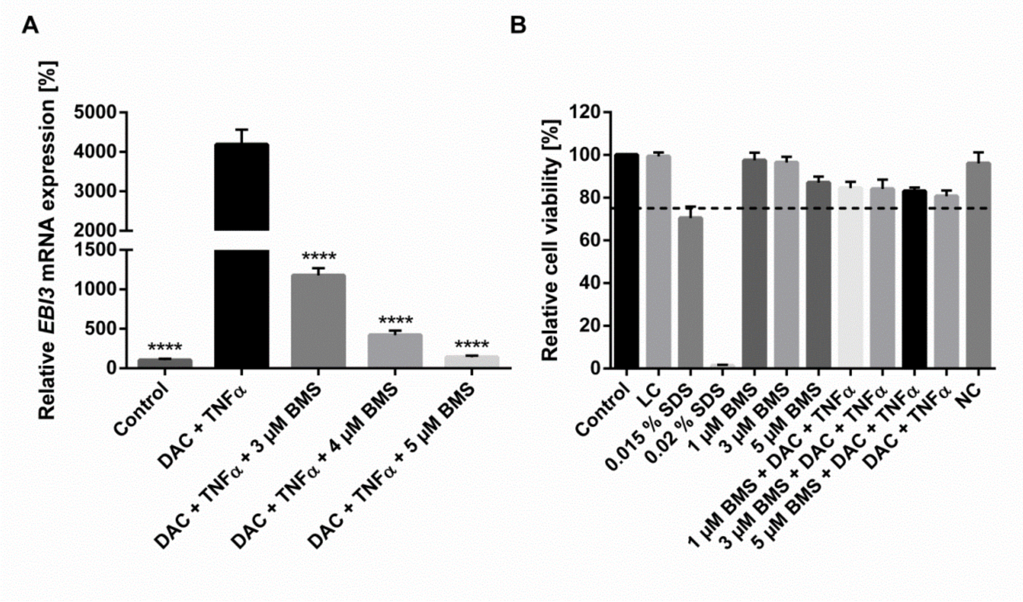

Supplementary Figure S4. BMS-345541 dose-dependently inhibits the synergistic induction of *EBI3* levels by DAC and TNFα. **(a)** HCEC cells were pre-treated with BMS-345541 (3, 4 or 5 μM) for 1 h before the stimulation with DAC (10 μM) or vehicle for 48 h with the addition of TNFα (20 ng/ml) for the final 24 h. The mRNA expression of *EBI3* was measured with RT-qPCR. *GAPDH* served as reference gene. Data are shown as mean ± SEM from three independent experiments. The differences in *EBI3* mRNA level compared to the DAC- and TNFα-stimulation were statistically analyzed by one-way ANOVA and Tukey's post hoc test (****$p < 0.0001$). **(b)** HCEC cells were treated with the indicated concentrations of BMS-345541, DAC (10 μM) and TNFα (20 ng/ml) for 48 h. TNFα was added for the final 24 h. Cell viability was determined by MTT assay. The data are expressed as percentage of the untreated control and are shown as mean ± SEM from three independent experiments.

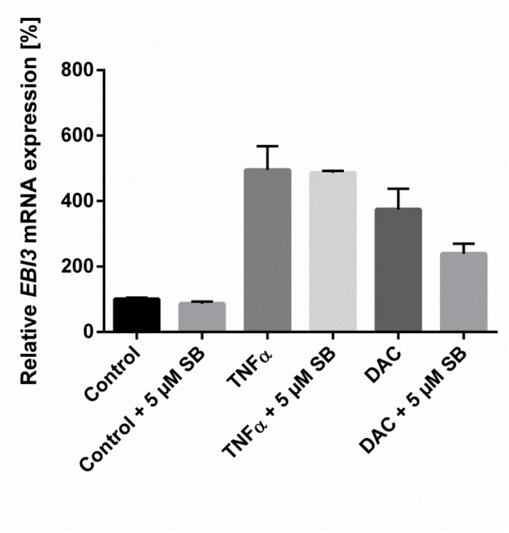

Supplementary Figure S5. The effects of p38 MAPKi on DAC- and TNFα-induced *EBI3* expression. HCEC cells were pre-treated with p38 MAPKi SB203580 (5 μM) for 1 h before the stimulation with DAC (10 μM) or vehicle for 48 h with or without the addition of TNFα (20 ng/ml) for the final 24 h. The mRNA expression of *EBI3* was measured with RT-qPCR. *HMBS* served as the reference gene. Data are shown as mean ± SEM from three independent experiments. Statistical analysis was performed using one-way ANOVA and Tukey's post hoc test.

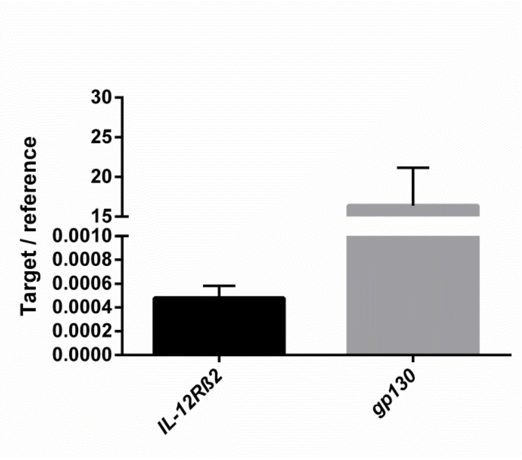

Supplementary Figure S6. Basal expression of IL-35 receptor subunits in HCEC. The mRNA expressions of *IL-12Rß2* and *gp130* in unstimulated HCEC were determined by RT-qPCR. *HMBS* served as the reference gene. Data are shown as mean ± SEM from three independent experiments.
